# Precipitation Characteristics of the Metastable γ″ Phase in a Cu-Ni-Be Alloy

**DOI:** 10.3390/ma11081394

**Published:** 2018-08-09

**Authors:** Zhiyuan Zhu, Yuanfei Cai, Yi Sui, Kexing Song, Yanjun Zhou, Jiasheng Zou

**Affiliations:** 1 School of Materials Science and Engineering, Jiangsu University of Science and Technology, Zhenjiang 212003, China; yzcaiyuanfei@126.com (Y.C.); cranescy@163.com (Y.S.); zjzoujs@just.edu.cn (J.Z.); 2 School of Materials Science and Engineering, Henan University of Science and Technology, Luoyang 471023, China; kxsong@haust.edu.cn (K.S.); zhouyanjun@haust.edu.cn (Y.Z.)

**Keywords:** Cu-Ni-Be alloy, aging process, habit plane, γ″ phase

## Abstract

The precipitation sequence of a Cu-Ni-Be alloy is: α-Cu supersaturated solid solution → Guinier-Preston (G.P.) zones → metastable γ″ → γ′ → stable γ (NiBe) phase. The micro-hardness and electrical conductivity during the aging process were measured. The precipitation characteristics and the distribution of the γ″ phase, under peak aging conditions, were analyzed by X-ray diffraction (XRD), transmission electron microscopy (TEM), selected area diffraction pattern (SADP), and high-resolution transmission electron microscopy (HRTEM). The results show that the orientation relationship of the γ″ phase/α-Cu matrix is: (001)*_p_*//(001)*_α_*; [100]*_p_*//[110]*_α_* (*p*: Precipitates, α: α-Cu supersaturated solid solution), which is in accordance with the Bain relationship in a FCC/BCC (face centered cubic/body centered cubic) structure, with the unique habit plane being {001}_α_. While the zone axis is parallel to [001]_α_, three forms of γ″ phases are distributed on the projection surface at the same time. The (001) reciprocal-lattice positions of γ″ phase in SADP are diffusely scattered, which is consistent with the variation of the *d*_(001)_ value of the γ″ phase. The intra-range variation is related to the distortion of the (001) plane of the γ″ phase, due to interfacial dislocations and distortion strain fields. The lattice of the γ″ phase in the HRTEM images was measured as a = b = 0.259 ± 0.002 nm and c = 0.27–0.32 nm. With the increase of thermal exposure time, the stable γ phase has a NiBe phase structure (Standard Card Number: PDF#03-1098, a = b = c = 0.261 nm), and the long diffuse scattering spots will transform into single bright spots. The edge dislocation, generated by interfacial mismatch, promotes the formation of an optimal structure of the precipitated phase, which is the priority of growth in the direction of [110]*_p_*.

## 1. Introduction

Beryllium copper alloys have many desirable properties, including good mechanical properties, good electrical conductivity, low elastic modulus, high wear resistance, and they are nonmagnetic. They also have special characteristics, including lack of sparks during the physical impact process, and they have been widely used in the automobile industry, aerospace, nuclear power, and many other areas [1,2,3]. They are typical of Cu-based alloy systems with a precipitation hardening effect, due to the formation of nanoscale Cu-Be, Ni-Be, or Co-Be precipitates in the Cu matrix upon solution and ageing heat treatment [4,5]. Beryllium prominently affects the properties of Cu-Be alloys, and alloy strength increases with an increase in beryllium content, while electrical and thermal conductivities decreases [1,3,5]. The physical properties of materials are determined by their microstructure construction, especially for the strengthening particles’ structure, scale, and crystallographic orientation relationship with the matrix. Many investigations have been reported in the phase transformation process during the aging process of Cu-Be-(Ni, Co) alloys; the precipitate sequence is believed to be an α-Cu supersaturated solid solution → Guinier-Preston (G.P.) zones → metastable γ″ ordered phase → γ′ → stable γ phase [6,7,8,9,10]. Cu-Be binary alloys in the process of the precipitated phase tend to undergo plane transformation into the metastable phase, which are {001}*_α_*, ({112}*_α_*, {113}*_α_*), and {111}*_α_*, respectively. The specific transformation mechanism is always being the focus of researchers. Monzen et al. [11] described the continuous precipitation process of a Cu-0.9 mass% Be single crystal as G.P. zones → γ″ → γ′_I_ → γ_I_ + γ′ → γ phase. The γ″, γ′_I_, and γ_I_ phases maintained Bain relationship with the matrix, i.e., ${\left(001\right)}_{\alpha }//{\left(001\right)}_{\gamma \prime \prime };{\left[110\right]}_{\alpha }//{\left[100\right]}_{\gamma \prime \prime }$, and the habit plane was {001}*_α_*. As the exposure time was prolonged, the habit plane of the metastable γ′ phase transformed to {112}*_α_* or {113}*_α_*, and the stable γ (CuBe) phase’s habit plane become {111}*_α_*. Geisler et al. [12] pointed out that the γ phase and copper matrix keep a N-W (Nishiyama-Wassermann) orientation relationship, i.e., ${\left(111\right)}_{\alpha }//{\left(110\right)}_{\gamma };{\left[1\bar{1}0\right]}_{\alpha }//{\left[001\right]}_{\gamma }$. The Cu-Ni-Be alloy aging precipitation process is similar to the Cu-Be alloy. In the early aging process, it forms disk-shaped monolayers of G.P. zones, which are made up of Be atoms that are parallel to {001}*_α_* of the Cu matrix. With the extension of heat preservation time, it forms disc shape structure by alternation and overlaying between the Be layer and the Ni layer. Watanabe et al. [13] define the γ″ phase as a disc shape structure with two to eight layers of Be atoms. The γ′ phase has 10 to 20 layers of Be atoms, and the γ phase’s atomic layer is more than 24 layers. The stable γ phase has a NiBe structure, and in the aging precipitation process, the precipitated phase always basically keeps a Bain orientation relationship with the matrix, i.e., the habit plane is always {001}*_α_*. The metastable phase’s transformation process is characterized by the increase of the thickness of the disc. The γ″, γ′ phase has a BCT (body-centered tetragonal) structure, with Ni atoms in a central position, and the crystal lattice constant being a = b < c. Furthermore, many investigations have reported that additional stress or severe deformation could influence the mechanical properties of high-conductivity and high-strength copper alloys. Cold deformation treatment, before the aging process, can promote the precipitation process [14], and the applied pressure stress to samples, during the aging process, can influence the distribution of G.P. zones in the matrix [15,16]. Ultra-fine copper alloy grain size can further improve the strength and conductivity of the precipitation strengthening alloy, which can be achieved by ARB (accumulative roll bonding) process [17,18].

Few investigations focus on the interfacial structure of the precipitate particles and the matrix, including their orientation, size, and shape. The objective of the present work is to focus on the precipitation behavior, including the phase morphology, size, and orientation of the phases, presented in the Cu-Ni-Be alloy during aging at 470 °C for different holding times, by XRD, TEM, and HRTEM. Meanwhile, the effect of precipitation on the properties of the aged Cu-Ni-Be alloy are studied as well.

## 2. Materials and Methods

Cu-Ni-Be alloy was melted by a medium frequency vacuum induction furnace. The raw materials were electrolytic pure Cu (99.99 wt.%), Cu—30 wt.% Ni, and Cu—3.3 wt.% Be intermediate alloy. The raw materials were melted at a temperature range from 1190 to 1210 °C, and the molten metals were poured into a metallic mold of cast iron to obtain an ingot. After subsequent hot-forging, thermal extrusion and cold drawing were performed to obtain a bar with a diameter of 16 mm. After chemical analysis, the composition the Cu-Ni-Be specimen was obtained, and is listed in Table 1. The specimens were solution-treated at 980 °C for 60 min, and then quenched in water, followed by aging at 470 °C holding for 10 min, 30 min, 1 h, 3 h, 8 h, 24 h, and 48 h respectively. Hardness was measured out using an automatic micro hardness machine (KB 30S, KB Prüftechnik, Hochdorf-Assenheim, Germany) under an indentation load of 5 kg for 15 s, and the electrical conductivity of specimens was measured using a Eddy Current Conductivity device (FQR-7501, Xingsha, Xiamen, China) according to the standard ASTM E1004-02 entitled “Standard Practice for Determining Electrical Conductivity Using the Electromagnetic (Eddy-Current) Method”. The metallographic image of the specimen was taken by an optical microscope (ZEISS, Oberkochen, Germany), with the specimen being pre-etched by HCl + FeCl_3_ + H_2_O solution. The specimen’s phase constitution was identified through XRD analysis (XRD 6000, Shimadzu, Kyoto, Japan) using Cu Kα radiation, and the specimen was scanned over a 2θ range from 35° to 105° with 2°/min.

For the observation of TEM and HRTEM, samples were cut to 0.5 mm wafers by electric discharge machining (EDM, LOMGKAI, Suzhou, China) from the peak aged alloy. Next, the samples were manually ground using SiC waterproof abrasive paper from 800 grit to 2000 grit to achieve a depth of 80–100 μm. Furthermore, the samples were punched to 3-mm diameter foils, and the foils were thinned to ~50 μm manually by 4000 grade abrasive paper. The foils were then twin-jet electro-polished in an electrolyte of nitric acid + methanol (with a volume ratio of 1:4) with a working current 60 mA at −30 °C. Before the observation in TEM, the polished foils were ion polished at 4 kV with a Gatan 691 with 4° ion incident angle for 30 min. The observations of the thin foils were carried out in a JEM 2100 transmission electron microscope (JEOL, Tokyo, Japan) at operating voltages of 200 kV, bright field image (BF), dark field image (DF) and selected area diffraction pattern (SADP) of the different electron beam incident axis were used to investigated the microstructure of the copper alloy specimen. Fast Fourier transform (FFT) and inverse fast Fourier transform (IFFT) techniques were used to analyze the HRTEM images by Digital Micrograph software (Version 2.32, Gatan, Pleasanton, CA, USA), to research the three-dimensional lattice structure of the precipitated phase.

## 3. Results

### 3.1. Metallographic Structure and Performance Analysis

**Figure 1:**
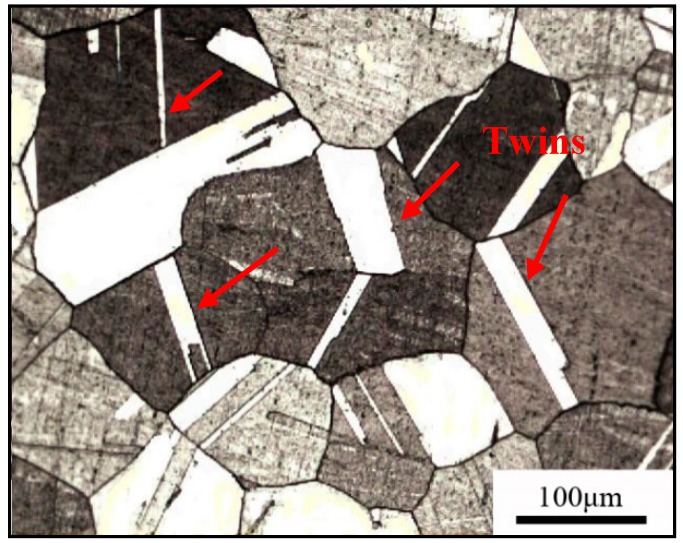
Optical micrograph of the Cu-Ni-Be alloy at the solution-aging state.

The optical micrograph of the Cu-Ni-Be alloy at the solution-aging state is shown in Figure 1; the aging treatment was 470 °C for 3 h, with a grain size distribution between 80 and 200 μm and distributed different forms of annealing twins in the matrix. In both sides of the twin crystal, an obvious difference of corrosion resistance was seen, which was mainly caused by atomic crystal column piling differences. A series of annealing twins were distributed on the grains, which were generated during the heat treatment process. The sample’s grain boundary shape was clear, and no discontinuous precipitation cellular structure was found.

**Figure 2:**
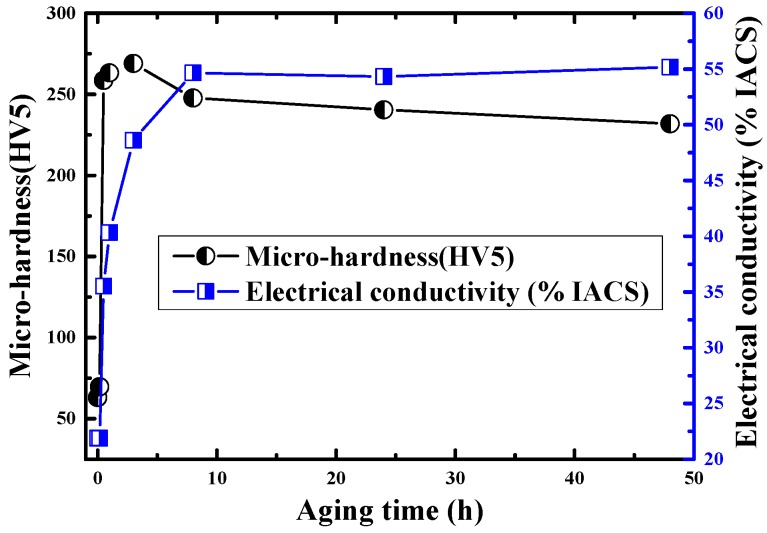
Micro-hardness and electric conductivity of the Cu-Ni-Be alloy at 470 °C as a function of aging time.

Figure 2 shows micro-hardness and electric conductivity of Cu-Ni-Be alloy at 470 °C during the aging process. At 470 °C during the aging process, the alloy’s electrical conductivity was increased. After heat preservation for 8 h, the alloy’s conductive rate reached around 55% IACS (International Annealed Copper Standard) and tended to be stable, while the solute atoms’ precipitation tended to be balanced. The change rule of microhardness of the alloy was: during early aging, the alloy strength grows with an approximate linear rule; with heat preservation for 30 min, the sample’s hardness is four times its solid solution state. During heat preservation for 3 h, the alloy’s hardness reached to the maximum value (269 HV5) and the corresponding conductivity was 48.6% IACS. Subsequently, the hardness of the sample was gradually reduced.

In the solid solution water cooling process, most of the solute atoms were solid and soluble in the Cu matrix, forming a supersaturated α-Cu solid solution; meanwhile they maintained a space equilibrium concentration under high temperature and formed a supersaturated vacancy. Matrix lattice distortion is very serious, as it sharply increases the electron scattering effect, and this shows the lowest conductivity. At the same time, the solute atoms’ solid solution effect was far less than the strengthening effect of the precipitation phase. In the early aging period, the supersaturation degree of the solute atoms in the α-Cu matrix was the highest, and with numerous open defects in the matrix. By space migration, the solute atoms first moved to the α-Cu matrix’s <100> soft elastic direction and forms a single disc shape G.P. zones structure [11,13,19], which kept a completely coherent relationship with α-Cu and produced an elastic coherency strain field, with the strength of the alloy increasing rapidly with it. The conductivity increases rate was smaller than the hardness growth rate. During heat preservation, after 30 min, the alloy sample’s hardness growth slowed; after extension of heat preservation to 3 h, the alloy’s hardness reached peak value. In this process, the G.P. zone structure growth had a structure of a multilayer disc shape γ″ phase or γ′ phase. This paper focuses on the precipitation strengthening phase orientation relationship of the Cu-Ni-Be alloy matrix under peak aging conditions.

### 3.2. X-ray Diffraction Analysis

**Figure 3:**
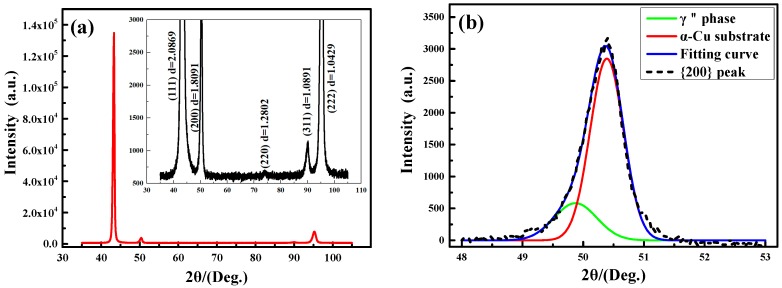
X-ray diffraction (XRD) analysis of the Cu-Ni-Be sample at an aging state of 470 °C for 3 h, (**a**) full-scale XRD pattern, (**b**) fitting curve of the {200} diffraction peak.

Figure 3 shows the XRD spectrum of the Cu-Ni-Be alloy in the peak aging state, and the diffraction intensity of the {111} crystal plane peak is extremely higher than other peaks. The enlarged view of the low diffraction intensity peak is shown in the inset of Figure 3a, and the corresponding inter-planar distance values are listed in the figure. The deviation of the actual inter-planar distance values and the standard pure-copper inter-planar distance values in the MDI Jade 6.0 database are listed in Table 2. The aging sample’s inter-planar distance values had deviations to the standard values, which was related to the alloy composition (content of solute atoms), heat treatment state (solid solution and aging state), surface residual stress, and equipment measurement error, etc. [20]. Under the same conditions, the interplanar spacing values of {200} and {220} peaks both showed a significant positive deviation (the *d* value is larger than the normal state), that is, the abscissa of the corresponding diffraction peak shifted to a low angle direction of the 2θ coordinate. This phenomenon indicates that the peaks of {200} and {220} were composed of two or more diffraction peaks. Considering the precipitation process of the Cu-Ni-Be alloy, we can identify that the precipitation phases formed on the {200} and {220} crystal planes of the Cu matrix, which induced the deviation of the peaks. The peaks of {200} and {220} were composed of two kinds of diffraction spectrum, including the {200}*_α_* and {110}*_p_*. The {200} diffraction peak could be decomposed into a low-angle γ″ phase peak and a high-angle α-Cu phase peak, which was fitted by Origin 2016 software (OriginLab, Northampton, MA, USA). The fitted curve is shown in Figure 3b, with the diffraction intensity of the γ″ phase being significantly lower than the diffraction intensity of the α-Cu phase, indicating that the content of precipitation phase in the matrix was very low.

### 3.3. Precipitated Phase TEM and SADP Analysis

**Figure 4:**
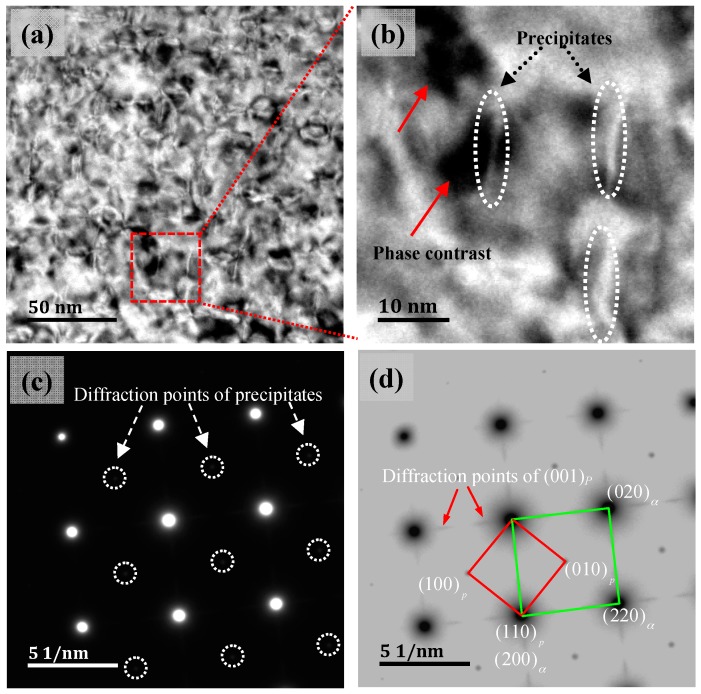
Transmission eletrcon microscopy (TEM) images of precipitates and the beam direction along [001]_α_, (**a**) bright field (BF) image, (**b**) the enlarged image of (**a**,**c**,**d**) the corresponding selected area diffraction patterns (SADPs), (**d**) is the inverse image of (**c**).

**Figure 5:**
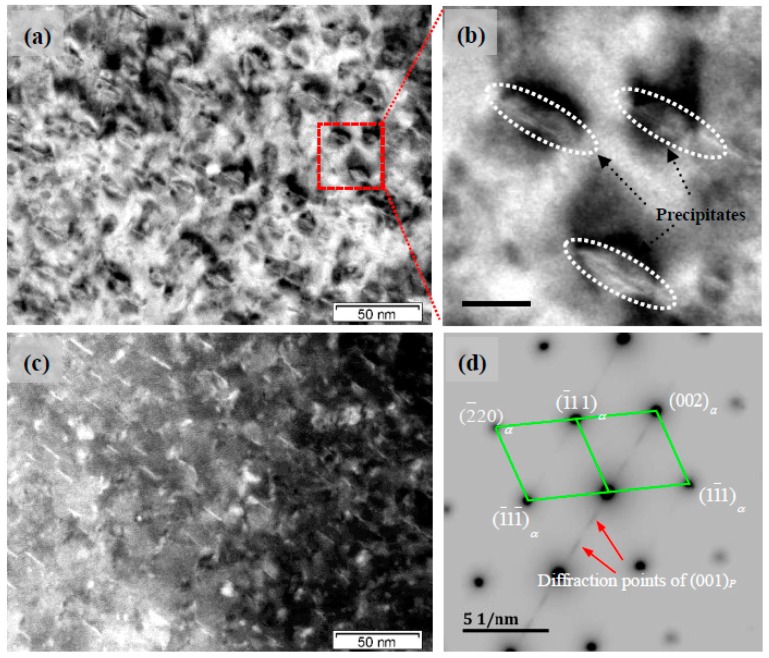
TEM images of precipitates, and the beam direction along [110]_α_, (**a**) the BF image, (**b**) the enlarged image of the (**a**,**c**) DF image, (**d**) the corresponding SADP image.

Figure 4 and Figure 5 show the TEM images of Cu-Ni-Be alloy at a peak aging condition of 470 °C for 3 h, and the electron beam direction parallel to [001]*_α_* and [110]*_α_*, respectively. High-density distortion strain field contrast could be observed in the BF images (Figure 4a and Figure 5a), and the precipitation particles were distributed on the α-Cu matrix uniformly. In a two-dimensional plane, it was characterized by the short rod, the length being about 10 nm. In the precipitated phase center, there was no contrast line feature and there was obvious distortion around the phase of the matrix. In TEM bright field conditions, it was characterized by a disc shape strain field contrast, as shown in Figure 4b and Figure 5b. Under [001]*_α_* incident conditions, we could clearly observe two forms of the precipitated phase, distributed at 90°. In the corresponding SADP diagram, the precipitated phase’s diffraction spots were distributed in the position of a reciprocal-lattice ban diffraction point with a face-centered cubic arrangement, 1/3 {002}_α_ and 2/3 {002}_α_. Diffraction spots of the band diffraction position were determined: ${\left(110\right)}_{p}//{\left(100\right)}_{\alpha };{\left[001\right]}_{p}//{\left[001\right]}_{\alpha }$. In the position of 1/3{002}_α_ and 2/3{002}_α_, the long strips of diffused scattering diffraction spots were formed by a (001) crystal plane in the precipitated phase. This shows that under the conditions of an incident electron beam, precipitated phase on the plane had a variety of forms [110]_α_, under the condition of incidence, only existed in one precipitated phase which distributed along a single direction. This related to the precipitated phase’s space distribution in the α-Cu matrix. In Figure 5d, the precipitated phase’s long strips diffused the scattering diffraction spot as in the TEM dark-field image shown in Figure 5c, where the precipitated phase was bright white, the distortion strain-filled contrast disappeared, and the matrix was black; in the corresponding SADP diagram, the precipitated phase’s diffraction spot position was in accordance with Figure 4d. Long strip scattering diffraction spots of the precipitated phase are related to the crystallography constant value.

### 3.4. γ″ Phase HRTEM Structure Analysis

**Figure 6:**
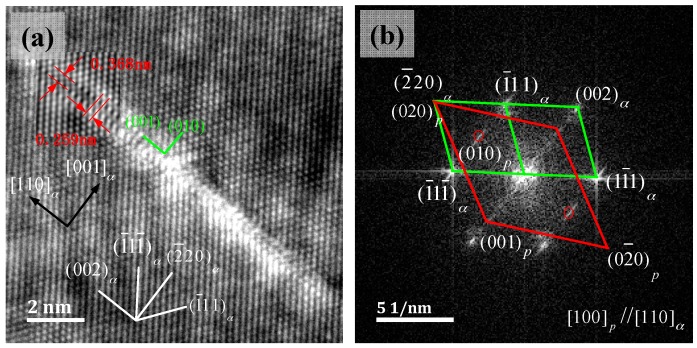
TEM image of a single γ″ phase, beam direction along [110]_α_, (**a**) HRTEM image, (**b**) the corresponding FFT reciprocal-lattice image.

**Figure 7:**
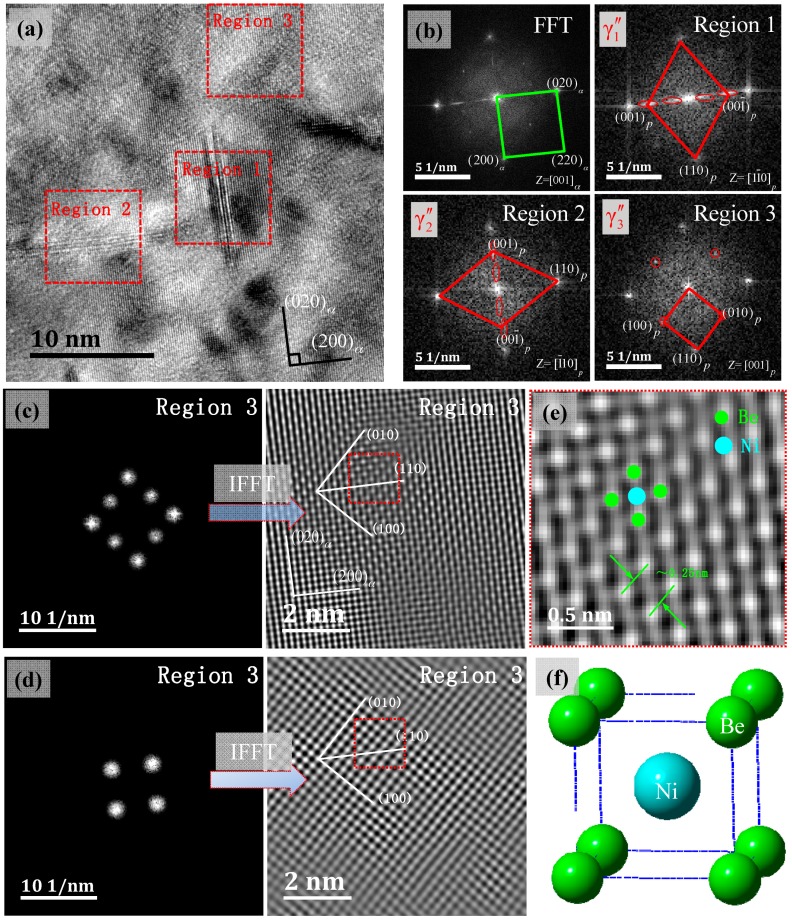
HRTEM, FFT and IFFT images of metastable γ″ phase, beam direction along [001]_α_, (**a**) three morphological γ″ phases, (**b**) FFT reciprocal-lattice images corresponding to different regions in (**a**,**c**), IFFT transformation image of region 3 in (**a**), with four pairs of diffraction spots {100} and {110} planes marked; the red frame shows the complete structural features of the precipitate, (**d**) IFFT transformation image of region 3 in (**a**), with two pairs of diffraction spots, {100} planes were marked, (**e**) the enlarged view of the red framed region in the (**c**), corresponding to a γ″ phase that is perpendicular to the paper, with the orientation of [001]*_p_*//[001]_α_, (**f**) the atomic unit-cell image of the stable NiBe phase.

Figure 6 shows a single γ″ phase HRTEM image and the corresponding FFT reciprocal lattice figure. Compared with the macroscopic SADP figure, added diffraction spots were present in the 1/2 {220}_α_ position. The long strip γ″ phase was parallel to the α-Cu matrix’s {002}_α_, and the orientation relation was described as: ${\left(001\right)}_{p}//{\left(001\right)}_{\alpha };{\left[100\right]}_{p}//{\left[110\right]}_{\alpha }$, and also existing as ${\left(010\right)}_{p}//{\left(\bar{1}10\right)}_{\alpha }$. This orientation relation was in line with the classic Bain relation of the FCC/BCC structure. [001]_α_ in the incident condition’s orientation relationship had a geometric equivalent with the Bain relation. The γ″ phase stable structure was an ordered NiBe phase with L1_2_ or B_2_. Ni atoms occupied the position of the body heart, the Be atoms were in the eight vertices, and unit cell structure was, as shown in Figure 7f, a metastable γ″, γ′ phase with a BCT structure, with a lattice constant: a = b < c. In the Figure 6a HRTEM diagram, the measurement location *d*_(010)*p*_ = 0.259 nm, *d*_(001)*p*_ = 0.368 nm, and the inter-planar distance of the γ″ phase (001) crystal plane’s two layers of Be atoms and the α-Cu matrix’s inter-planar distance were consistent. However, in the corresponding FFT reciprocal lattice diagram, (002)*_p_* and (002)*_α_* diffraction point locations were not coincident, which means that *d*_(001)*p*_ < 0.368 nm, and the actual measured value was between 0.27 nm to 0.32 nm, Ken’ichi et al. [21] measured the lattice constant of the γ″ phase of the Cu-Ni-Be alloy to be a = b = 0.253 nm, c = 0.29 nm and Watanabe et al. [13] measured the lattice constant of the γ″ phase of the Cu-Ni-Be alloy to be a = b = 0.24 nm, c = 0.28 nm. Two layers of the structure of the γ″ phase were imaged by TEM, showing that its diffraction intensity was weak and vulnerable to interference by the α-Cu matrix’s diffraction wave, as shown in Figure 5d. The 1/2 {220}_α_ position did not show obvious γ″ phase diffraction spots, while these appeared in the Figure 6b FFT reciprocal lattice. In HRTEM, the γ″ phase position showed a phase contrast that was significantly higher than the surrounding matrix; because the atomic number of Be is small, it has a small influence on the transmission wave’s transfer function and appears as torsion of the lattice fringe.

Figure 7 shows the γ″ phase in the condition of [001]*_α_* incidence, the HRTEM and FFT reciprocal lattices, and the IFFT transformation image of the corresponding areas. In Figure 7a there were obviously two mutually perpendicular γ″ phases; they were respectively parallel to the α-Cu matrix’s (020) and (200) crystal planes. At the same time, there was a class of the γ″ phase that was parallel to [001]*_α_* and perpendicular to the projection plane. The precipitation position of this orientation γ″ phase required a FFT change to be determined. At the γ″ phase position, an obvious ordered lattice reflection point appeared, and while the matrix position did not appear. Figure 7b shows different areas of the FFT image in Figure 7a, respectively, including the whole region and regions 1, 2, and 3, and they were in three correspondingly different distribution forms of the γ″ phase in the α-Cu face-centered cubic crystals, while the γ″ phase’s habit plane was {001}*_α_*. The three states determined the following orientation relations of the γ″ phase in the α-Cu matrix, i.e., three variants:

$$\mathrm{Variant}A\left(\gamma_{1}^{\prime \prime }\right):\;{\left(110\right)}_{p}{//(100)}_{\alpha };[1\bar{1}{0]}_{p}{//[001]}_{\alpha };$$

$$\mathrm{Variant}B\left(\gamma_{2}^{\prime \prime }\right):\;{\left(110\right)}_{p}{//(010)}_{\alpha };[1\bar{1}{0]}_{p}{//[001]}_{\alpha };$$

$$\mathrm{Variant}C\left(\gamma_{3}^{\prime \prime }\right):\;{\left(110\right)}_{p}{//(100)}_{\alpha }{;[001]}_{p}{//[001]}_{\alpha }.$$

**Figure 8:**
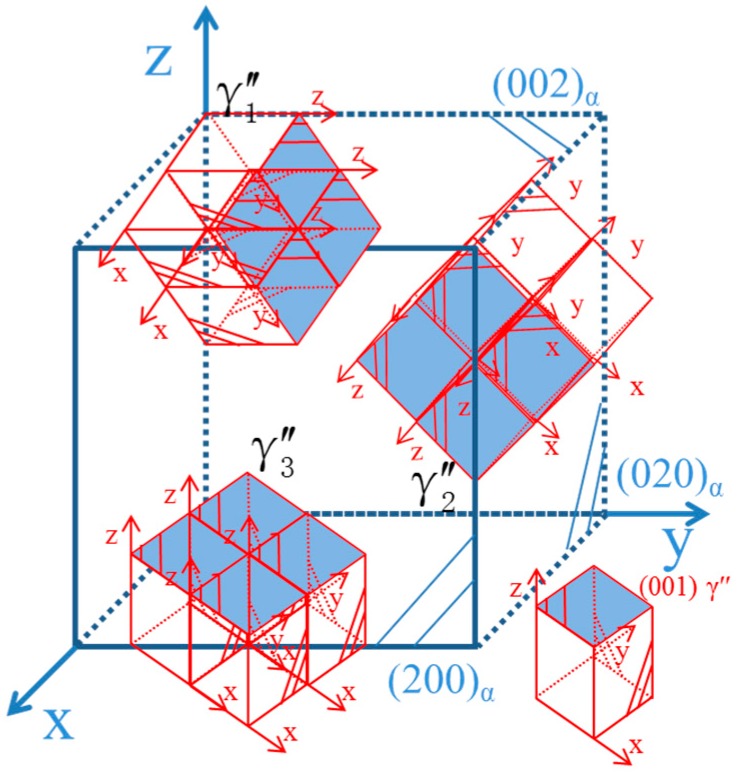
Schematic graph of the γ″ phase spatial distribution in the Cu cubic lattice, γ″ phase unit cell size: a = b < c.

Yonghua et al. [22] studied the γ″-Ni_3_Nb precipitated phase’s spatial distribution pattern in a FCC matrix of Inconel 718 alloy. The {100}_γ″_//{100}_γ_; [001]_γ″_//<100>_γ_, γ″-Ni_3_Nb had a D0_22_ ordered structure, and also had three variations of orientational relationship. The Cu-Ni-Be alloy’s γ″ phase’s spatial distribution to form the Cu matrix is shown in Figure 8; they respectively corresponded to the above three orientation relationships of the variants. As shown in Figure 7c, (100)*_p_*, (010)*_p_*, (200)*_α_* and (020)*_α_* were chosen for IFFT imaging and could be modulated and enhanced as a two-dimensional structure. In Figure 7c, (100)*_p_* and (010)*_p_* were chosen for IFFT imaging and could be modulated into a two-dimensional lattice image. In Figure 7c, lattice the transition zone could be observed in the surrounding γ″ phase. The γ″ phase had a B2 type ordered structure characterization (a = b < c) and a Ni atom located in the center of the matrix. In the condition of [001]*_p_* incidence, the a = b, γ″ phase and the α-Cu matrix interface feature was the FCC/BCC lattice transformation. The orientation relationship was: ${\left(110\right)}_{p}//{\left(100\right)}_{\alpha };{\left[001\right]}_{p}//{\left[001\right]}_{\alpha }$, and its relationship with Bain relation was the geometric equivalent. In HRTEM–FFT-IFFT, the image is represented as a bright dot contrast. In region 3, γ″ assumed two-dimensional extended forms, i.e., the γ″ phase had a disc shape form. The disc-shaped γ″ phases preferred to broaden along the [100]*_p_* and [010]*_p_* directions (equal to the [110]*_p_* direction), rather than grow along the [001]*_p_* direction.

**Figure 9:**
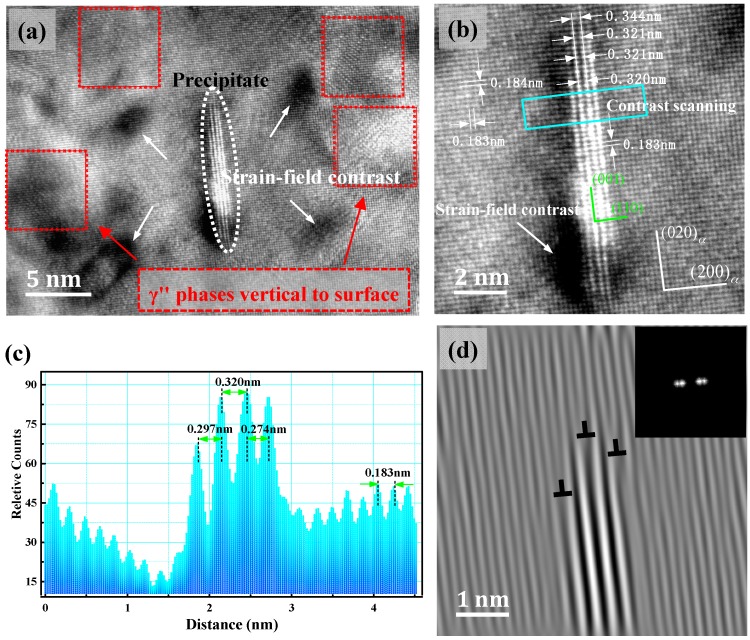
Interface structure of the γ″/Cu matrix and the distorted strain-field contrast, with a beam direction along [001]*_α_*, (**a**) HRTEM image, (**b**) the enlarged image of the γ″/Cu interface structure, (**c**) phase contrast image of the region across the γ″ phase in (**b**), which was obtained by Digital Micrograph software, (**d**) IFFT filter image of the γ″/Cu interface region, the inset graph showing diffraction points marking the status.

**Figure 10:**
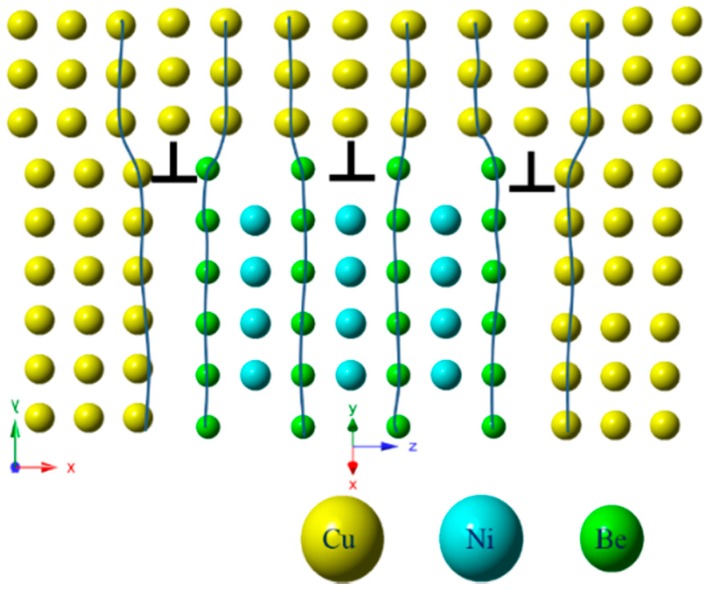
The unit-cell simulation diagram of the γ″/Cu matrix interface; γ″ phase unit cell size: a = b < c.

Figure 9 shows a distorted strain contrast HRTEM image of the γ″ phase. With the FFT method to determine in Figure 9a, in the red square area, there were γ″ phases which were perpendicular to the projection plane. In the 5–10 nm range of the γ″ phase, α-Cu matrix’s lattice generated an elastic deformation, which affected the transmission and diffraction transfer function in the matrix. The HRTEM images showed the weak phase contrast. Figure 9b shows the γ″/Cu interface morphology. The middle two layer distances of the Be atom, i.e., *d*_(001)*p*_, gradually reduced from the interface edge of 0.344 nm, to 0.320 nm. In Figure 9c, the measured distances between the four layers were 0.297, 0.320, and 0.274 nm respectively, i.e., *d*_(001)*p*_ did not have a definite value, but changed within a certain range. This result was well explained as the (001)*_p_* crystal plane’s diffraction point in reciprocal space was a stripe, and a diffuse scattering point formed. It extended along the {002}*_α_* crystal plane direction of the matrix. In the late aging precipitation period, the γ″ phase transferred to a metastable γ′ phase and a stable γ phase. The (001)*_p_* reciprocal space diffraction spot shrank to a point [20,23], while *d*_(001)*p*_ tended to a stable value. The Stable γ-NiBe had a cubic structure, a = b = c = 0.261 nm (PDF#03-1098). The FFT transform was performed on the γ″/Cu interface region and two pairs of diffraction spots for the γ″ phase and the α-Cu matrix phases were chosen for FFT-IFFT transform. The result is shown in Figure 9d, and it is can be clearly observed that three groups of edge dislocations were distributed on the γ″/Cu interface, with the Burgers vector direction being parallel to (200)*_α_*. The emergence of the interface dislocation significantly reduced the interface elastic strain energy of the two phases of γ″/Cu, and the interface energy increased, reducing the energy of the system. Based on Figure 9d, the γ″/Cu interface cell simulation diagram was built, (Figure 10, ${\left[1\bar{1}0\right]}_{p}//{\left[001\right]}_{\alpha }$), with a *d*_(001)*p*_ value between 0.27 nm and 0.32 nm, and greater than *d*_(020)*α*_ 0.184 nm(actual measured value). According to the formula of mismatch degrees [24]:

(1) $$\varepsilon =2\frac{d_{p}-\;d_{\alpha }}{d_{p}+d_{\alpha }}\;$$

In Formula (1), *d_p_* and *d_α_* correspond to the inter-planar distance of the precipitated phase and matrix respectively; *ε* is the interface mismatch value between the precipitate and matrix. The calculated interface mismatch of *d*_(001)*p*_ and *d*_(020)*α*_ was between 0.38 and 0.54, which could not keep a coherent orientation relationship. In the interface, mismatched edge dislocation was generated in order to reduce the elastic strain energy of coherency. In the interface dislocation position, the elastic strain energy is small and the solute atoms easily spread to the interface dislocation position. Therefore, the γ″ phase preferred to grow as a disc shape along [110]*_p_*, and the growth in the [001]*_p_* direction was controlled. The interface mismatched dislocation’s formation could promote the precipitated phase to form a preferred structure [25]. In the γ″ phase growth process, climbing of the interface edge dislocation occurred, the movement of which was governed by the spread of the solute atoms or the space. In the early period of aging, a supersaturation point’s defects rapidly promote the aging process. With the development of the aging process, the precipitated phase should be controlled by the diffusion of solute atoms and the migration process.

## 4. Analysis and Discussion

In the early aging period, the Cu-Ni-Be alloy’s hardness rapidly rises, and the increased rate of conductivity lags behind the growth rate of alloy hardness. This is because, in the early aging precipitation period, the G.P. zone’s structure size is very tiny (~0.1 nm), which can produce additional scattering on the electron and impact electron’s movement, and then slow down the increase of conductivity rate. The activation of formation of the G.P. zones is related to the spread of the point defect [26]. The retained heat balance point defect of the solid solution water cooling process is quickly spread in the early stages of aging heat preservation. This promotes the rapid formation of the G.P. zones. After the formation of G.P. zones, the metastable γ″ phase transforms to the γ′ phase and to the γ phase. This process is controlled by the Ni atom’s diffusion in the Cu matrix [13]. Research shows that the γ phase’s thickening rate is directly proportional to *t*^1/2^. The diffusion coefficient, D = 2.3 × 10^−20^ m^2^ s^−1^, is in one order of magnitude, with Ni atoms’ diffusion coefficient being D = 5.3 × 10^−20^ m^2^ s^−1^ in Cu, and it is far smaller than the Be atoms’ diffusion coefficient 3.4 × 10^−18^ m^2^ s^−1^ [27] in Cu.

The Cu-Ni-Be alloy and the Cu-Be alloy shows very similar characteristics in the aging precipitation process, including the morphology of early G.P. zones and their formation mechanisms, as well as in their γ″ phase morphologies and crystal sizes. However, the former stable phase is the NiBe phase and the latter is the CuBe phase. These two stable phases have L1_2_ or B2 ordered structure characteristics, and the cell structure is similar to a body-centered cubic crystal. Therefore, we can use the FCC/BCC interfacial orientation theory to analyze the orientation relation between the two stable phases, the NiBe, CuBe, and the Cu matrix [28,29]. Based on the side-side matching model, we put forward the criterion of the FCC/BCC’s structural approach relationship:

*Β* = *a_F_*/*a_B_* (2)

**Figure 11:**
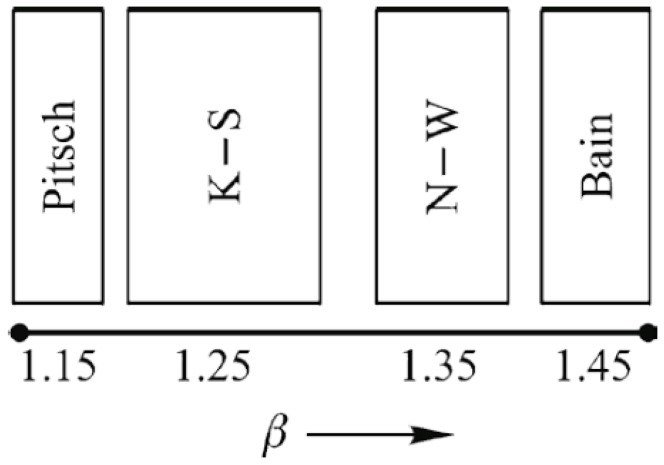
Favored orientation relationships predicted in the FCC/BCC system as the values of the lattice parameter ratio (K-S: Kurdjumov-Sachs, *β* = *a_F_*/*a_B_*) [28].

In Equation (2): *a_F_* is the lattice constant of the FCC crystal, *a_B_* is the lattice constant of the BCC crystal, and the *β* criterion is the ratio of *a_F_* and *a_B_*. The selected relevant crystallography data are: NiBe (PDF#03-1098): a = 0.261 nm, CuBe (PDF#31-0182): a = 0.273 nm, Cu (PDF#04-0836): a = 0.3615 nm, and respectively calculated *β_NiBe_* = 1.385, *β_CuBe_* = 1.324. The crystal’s parameter ratio, the *β* criterion, refers to Figure 11. It is known as *β_CuBe_* < 1.35 and *β_NiBe_* > 1.35. Thus, the γ-CuBe phase and the Cu matrix tend to form a N-W relation; the γ-NiBe phase and the Cu matrix tend to form a Bain relationship. Before forming a stable γ-CuBe phase, the Cu-Be alloy had a metastable γ″ phase a = b ≈ 0.25 nm, c ≈ 0.32 nm, which can basically keep a Bain relation with the Cu matrix, but as the precipitated phase coarsens, the crystallographic constant tends to a steady structure and the corresponding orientation relationship subsequently transforms to a N-W relationship, i.e., the habit plane is {111}*_α_*. However in a transition stage, two unstable habit planes appear, {112}*_α_* and {113}*_α_*. The Cu-Ni-Be alloy in the precipitation process does not exist this habit plane’s transformation, and the precipitated phase is always parallel to the Cu matrix’s {001} crystal plane. This structure is completely matched with the matrix lattice and may be the intrinsic reason for the Cu-Ni-Be alloy having a high conductivity. The γ″, γ′, and γ phases are in the Ni-Be alloy in the high conductivity Cu-Ni-Be alloy, which is the same as the structure of γ″, γ′, or γ phases (CuBe) in the high strength Cu-Be binary alloy. However, the Cu-Be alloy system possesses multi-habit plane precipitation phases, which can significantly improve the strength of the alloy. Distortion of the copper matrix lattice increases, and its scattering of electrons is bigger, and the electrical conductivity is relativity lower compared with the Cu-Ni-Be alloy.

## 5. Conclusions

The aging precipitation process of the Cu-Ni-Be alloy obeys the classical development mechanism, i.e., G.P. zones → metastable γ″ → γ′ → stable γ. The Electrical conductivity increases continually and the hardness value raises at the early stage and then descends. In about 2 h to 3 h, the specimen achieved peak hardness value. The precipitated phase’s growth was controlled by the diffusivity of Ni atoms in the Cu matrix.

- The metastable γ″ phase lattice constants in the present research is a = b = 0.259 ± 0.002 nm, with *d*_(001)*p*_ changes in the range of 0.27–0.32 nm. A (001)*_p_* diffuse scattering diffraction spot in the reciprocal space is the long trip shape, and with the increase of the metastable phase size, with *d*_(001)P_ tending to a stable value. Long strip diffraction spots will shrink to a dotted contrast.
- The orientation relationship between the γ″ phase and the α-Cu matrix is: ${\left(001\right)}_{p}//{\left(001\right)}_{\alpha };{\left[100\right]}_{p}//{\left[110\right]}_{\alpha }$, which is known as the Bain relation in the FCC/BCC structure. The Habit plane is {001}*_α_*. [001]*_α_* under the condition of incidence, and there are three forms of the precipitate phase in the matrix, and it is a disc shape. They distribute vertically with each other in the Cu matrix three-dimensional space. Interface mismatches will promote the precipitated phase to form an optimal structure. The γ″ phase prefers to grow along the [110]*_p_* direction.
- The Cu-Be and Cu-Ni-Be precipitated phase rule in the alloy precipitation conforms to the interface matching rule of the FCC/BCC structure. In the Cu-Be alloy, along with the aging process, the relationship between the precipitated phase and the matrix transforms from the Bain relationship to N-W, i.e., a habit plane transformation. The Cu-Ni-Be alloy aging precipitation process always maintains the Bain relationship and does not have a habit plane transformation. This depends on the crystallographic constant of the precipitated phase (CuBe, NiBe).

## Figures and Tables

**Table 1:** Chemical composition of the alloy.

Element	Ni	Be	Cu	Impurity
Contents (wt.%)	2.16	0.272	Bal.	≤0.1

**Table 2:** Deviation of the actual inter-planar distance and the standard copper inter-planar distance (unit: Å).

PDF Cards	{111}	Error	{200}	Error	{220}	Error	{311}	Error	{222}	Error
PDF#04-0836	2.088	−0.0011	1.808	0.0011	1.278	0.0022	1.09	−0.0009	1.0436	−0.0007
PDF#65-9026	2.086	0.0009	1.8065	0.0026	1.2774	0.0028	1.0894	−0.0003	1.043	−0.0001
PDF#65-9743	2.083	0.0039	1.8039	0.0052	1.2756	0.0046	1.0878	0.0013	1.0415	0.0014
PDF#70-3038	2.0929	−0.0060	1.8125	−0.0034	1.2816	−0.0014	1.093	−0.0039	1.0465	−0.0026
PDF#70-3039	2.086	0.0009	1.8065	0.0026	1.2774	0.0028	1.0894	−0.0003	1.043	−0.0001
PDF#85-1326	2.0871	−0.0002	1.8075	0.0016	1.2781	0.0021	1.09	−0.0009	1.0435	−0.0006
PDF#89-2838	2.0872	−0.0003	1.8075	0.0016	1.2781	0.0021	1.09	−0.0009	1.0436	−0.0007
Actual data	2.0869		1.8091		1.2802		1.0891		1.0429	
